# Distribution of pharmacy deserts and its association with digital divide and residential redlining across the United States

**DOI:** 10.1371/journal.pone.0330027

**Published:** 2025-08-11

**Authors:** Giovanni Catalano, Selamawit Woldesenbet, Timothy M. Pawlik

**Affiliations:** 1 Department of Surgery, The Ohio State University Wexner Medical Center and James Comprehensive Cancer Center, Columbus, Ohio, United States of America; 2 Department of Surgery, University of Verona, Verona, Italy; King Faisal University, SAUDI ARABIA

## Abstract

**Background:**

Recent pharmacy closures across the US has increased the number of communities characterized as “pharmacy deserts.” Residential segregation and structural economic disinvestment including the digital divide may exacerbate inequities related to pharmacy access.

**Methods:**

In this cross-sectional study, pharmacy deserts were defined at the census tract level and their distribution was analyzed relative to the digital divide index (DDI) and residential redlining using multivariate logistic regression.

**Results:**

Overall, 3,105 (3.72%) census tracts were classified as pharmacy deserts comprising more than 10 million inhabitants (n = 10,215,249). Pharmacy deserts were more often Black (n = 398, 13% vs. n = 6142, 7.6%), Hispanic (n = 597, 19.0% vs. 7662, 9.5%), or American Indian and Alaska Native (n = 82,14.0% vs. n = 113, 0.1%) segregated communities (all p < 0.001). Census tracts with a high DDI (OR 6.94, 95%CI 5.82–8.32; p < 0.001; E-value = 4.70) had a higher likelihood of being a pharmacy desert versus low DDI areas. Furthermore, census tracts experiencing high residential redlining had a twofold increased risk of being pharmacy deserts (OR 2.18, 95%CI 1.90–2.50; p < 0.001; E-value = 2.31).

**Conclusions:**

Understanding how socioeconomic and infrastructure factors influence access to pharmacies is crucial to reduce health inequities. Efforts should be made to ensure equitable access to pharmacy services, especially for underserved populations in both rural and urban settings.

## Introduction

Pharmacies play a vital role in the US health care system, as highlighted by the prevalence of prescription medication use across the country. In fact, about 60.0% of adults in the US took at least one prescription medication in 202 with 36.0% of adults taking three or more [1]. In addition, pharmacies offer a wide variety of health services such as helping patients manage complex medication regimens, reviewing drug interactions, performing health screenings, immunizations, and patient counseling, speaking to the integral role of pharmacies in supporting and enhancing patient medication adherence and overall health [2,3]. Moreover, the recent COVID-19 (coronavirus disease 2019) pandemic has further highlighted the importance of pharmacies as points of access for providing access to essential health services during times of public crises. Unfortunately, pharmacy closures have become an increasingly important issue in the US, with thousands of drugstore chain locations closing nationwide [4]. Therefore, similar to the concept of a “food desert,” the term “pharmacy desert” has been introduced to describe any geographic area in which medications may be harder to obtain [5].

Pharmacy deserts have been described as communities with impaired access to pharmacies, and are more prevalent in historically marginalized neighborhoods, leading to an increase in already-existing health inequities [6]. In fact, neighborhood characteristics such as socioeconomic status and walkability are closely related to inequities in access to healthcare, and residential segregation has been recognized as an important driver of health disparities among different racial and ethnic groups especially in urban settings [7,8]. This so-called “contemporary redlining” is the manifestation of structural racism and economic disinvestment, and persists through biased mortgage lending practices based on property location [9]. The coexistence of pharmacy deserts in redlined areas may exacerbate health inequities by limiting access to medications and basic healthcare services, leading to increased medication non-adherence and worse health outcomes among already disadvantaged communities. Furthermore, given the increasing number of pharmacy closures, telepharmacy has been proposed as a viable alternative to traditional physical pharmacies, and many states have implemented policies expanding telepharmacy practice [5]. However, the ability of patients to access telehealth services, especially in rural and socially disadvantaged areas, has been a growing concern [10]. The digital divide is defined as the gap between communities that have access to modern technology infrastructure versus residential areas that do not [11]. The potential impact of digital divide on health inequities has prompted significant policy discussions, leading the Federal Communications Commission (FCC) and other national organizations to define broadband access as a “super-determinant” of health, as it affects numerous other social determinants of health [12]. To measure this technology gap across different areas, the Digital Divide Index (DDI) was developed as a measure of broadband infrastructure and adoption and socioeconomic characteristics that may limit motivation, skills, and usage of digital means [13].

In this context, the aim of the current study was to analyze the distribution of pharmacy deserts across the US and to define the relationship between pharmacy deserts and social and structural inequalities such as residential redlining and the digital divide.

## Materials and methods

Data on community retail pharmacies across the US were sourced from the Homeland Infrastructure Foundation-Level Data (HIFLD) [14,15]. Data to define pharmacy deserts characteristics were sourced from the 2020 Decennial Census [16] and the 2021 5-year American Community Survey, [17] which contain data on population counts and estimates of social and demographic characteristics, including race/ethnicity (i.e., White, Black, Hispanic, Asian, AIAN [American Indian and Alaska Native]), education level, health insurance status, and median income. These tract-level datasets were merged using the unique 11-digits geographic identifiers. Communities were defined as segregated White, Black, Hispanic, Asian, or AIAN if more than 50.0% of the population was non-Hispanic white, non-Hispanic black, Hispanic, non-Hispanic Asian, or non-Hispanic AIAN, respectively. Communities where no single racial or ethnic group comprised a majority were classified as “integrated” [18,19]. Census tracts were categorized as urban, suburban, and rural based on previously defined population-density thresholds (i.e., urban: > 5,000 inhabitants/square-mile; suburban: 1,000–5,000 inhabitants/square-mile; rural: < 1,000 inhabitants/square-mile) [20,21]. The census tract-level Digital Divide Index (DDI) was used to measure access to modern digital infrastructure and technology adoption [13]. Specifically, DDI is composed of two separate scores: the Infrastructure/Adoption score (IFNA) and the Socioeconomic score (SE), each ranging from 0 to 100. The IFNA is calculated based on five variables related to broadband infrastructure and adoption, including the proportion of the population with broadband internet access, whereas the SE is calculated based on socioeconomic variables known to impact technology adoption, such as the proportion of younger and older inhabitants, poverty rate and income, high school education rate. The final DDI ranges from 0 to 100, with higher values indicating lower availability of modern information technology, and has been used in prior studies to assess access to healthcare. DDI scores were categorized into “low,” “moderate,” and “high” using quartiles [11,22,23].

Residential redlining was measured using the Residential Redlining Index (RRI), which represents the odds ratio of denial of a mortgage application for a property in a local area, compared with properties across the metropolitan statistical area (MSA) [9]. Values lower than one represented a lower likelihood for the mortgage to be denied, whereas values greater than one represented a higher likelihood for mortgage applications to be denied. Redlining scores were categorized as “low” (<0.7), “neutral” (0.7–1.3), and “high” (≥1.3) based on equal distances from 1.0 on the reciprocal scale, consistent with previously published literature, to reflect meaningful differences in mortgage denial odds [24,25]. This study was deemed exempt by the Institutional Review Board because only publicly available de-identified census tract-level data were used. The authors had no access to information that could identify individual participants during or after data collection. The STROBE statement was followed in reporting this study.

### Pharmacy desert definition

A census tract was defined as a pharmacy desert if it met both the “low-income” and “low-access” criteria, consistent with previous reports [6,21]. A census tract met the “low-income” designation if either (i) ≥20% of its population was below the Federal poverty level or (ii) the median household income was < 80% of the median income of the nearest MSA. The “low-access” criterion was met if a census tract ≥33% of its population living 1-, 5-, or 10-miles linear distance or more from the pharmacy for urban, suburban, and rural tracts, respectively. Furthermore, a census tract met the “low access” criterion if it had < 100 individuals owning a car and ≥33% of its population living 0.5 miles linear distance from the nearest pharmacy. The population living within the defined accessibility radius of a pharmacy was assessed using areal interpolation at the block level, evaluating whether the centroid of every census block in the US was inside or outside of the pharmacy accessibility radius. The proportion of a tract’s population living within the acceptable radius of a pharmacy was calculated dividing the sum of the population of the blocks that were inside a pharmacy accessibility radius by the total population of the tract.

### Statistical analysis

Continuous variables were summarized as median and interquartile range (IQR) and compared using the Wilcoxon rank sum test. Categorical variables were reported as frequencies and percentages (%) and compared using the Chi-square test or Fisher’s exact test, as appropriate. Logistic regression analyses were performed to assess relationships between the variables of interest, and results were reported as odds ratios (ORs) with 95% confidence interval (95%CI). Multicollinearity was assessed using the Variance Inflation Factor (VIF). Spatial autocorrelation was evaluated using Moran’s I statistics on the model-predicted odds. To address potential unmeasured confounding, E-values for the estimated ORs of the primary variables of interest were calculated. A two-sided P-value <0.05 was considered statistically significant. All analyses and geospatial data processing and visualizations were performed using the R programming language (version 4.3.2; R-Foundation, Vienna, Austria).

## Results

**Fig 1:**
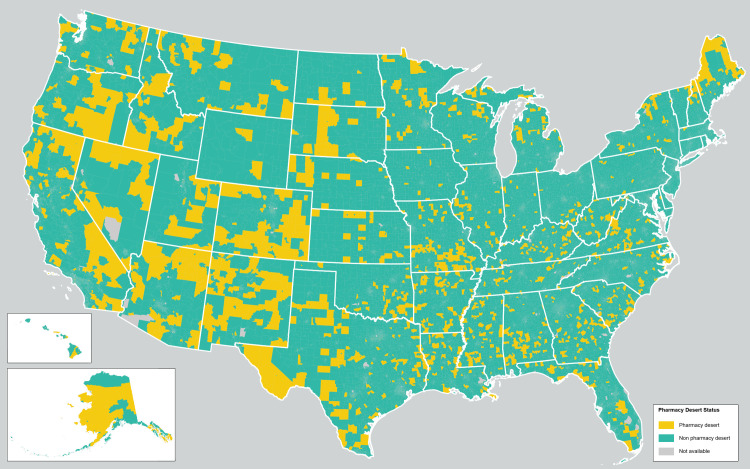
Geographic distribution of pharmacy deserts across the United States.

**Fig 2:**
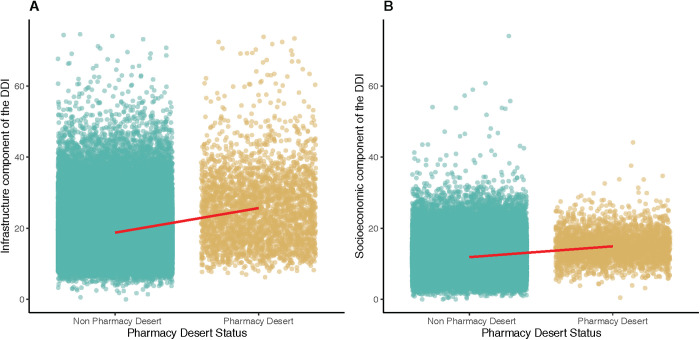
Association of pharmacy desert status and (A) the infrastructure and (B) socioeconomic component of the Digital Divide Index.

Among the 85,479 census tracts analyzed, 83,398 had data on population and income and were included in the analytic cohort. Overall, 3,105 (3.72%) census tracts were classified as pharmacy deserts, comprising more than 10 million inhabitants (n = 10,215,249) or about 3.1% of the US population (**Fig 1**). Pharmacy desert communities had a slightly lower proportion of female inhabitants (50.2%, IQR 48.9%−51.6% vs. 51.0%, IQR 49.9%−52.2%; p < 0.001) and adults over the age of 65 years (14.7%, IQR 8.7%−21.7% vs. 15.7%, IQR 11.2%−20.8%; p < 0.001) versus non-pharmacy desert communities. Urban and rural census tracts were more often designated as pharmacy deserts compared with suburban census tracts (p < 0.001). Pharmacy deserts were characterized by a higher proportion of inhabitants with high school education or less (34.6%, IQR 28.1%−40.7% vs. 28.1%, IQR 19.6%−35.7%), no health insurance (12.3, IQR 7.3%−19.9% vs. 7.6%, IQR 3.9%−13.4%), and ambulatory disability (10.4%, IQR 6.9%−14.4% vs. 7.6%, IQR 4.9%−11.1%) (all p < 0.001). Median income was lower in pharmacy desert communities versus non-pharmacy desert communities ($43,203, IQR $35,913-$50,885 vs. $63,718, IQR $47,356-$86,993; p < 0.001). Pharmacy desert communities were also more often comprised of Black (n = 398, 13% vs. n = 6142, 7.6%), Hispanic (n = 597, 19.0% vs. 7662, 9.5%), or AIAN (n = 82,14.0% vs. n = 113, 0.1%) patients (all p < 0.001). In addition, pharmacy deserts had a higher median DDI (25.9, IQR 20.4–31.4 vs. 18.6, IQR 14.1–23.9) with a more prominent increase in infrastructure development (24.6, IQR 17.8–31.6 vs. 17.3, IQR 13.4–22.8) versus socioeconomic components (14.6, IQR 12.2–17.3 vs. 11.4, IQR 8.8–14.4) related to the DDI score compared with non-pharmacy deserts (all p < 0.001) (**Fig 2**). Furthermore, pharmacy deserts were more likely to have a high redlining index (n = 1,057, 34% vs. n = 19,305, 24%) versus non-pharmacy desert communities (**Table 1**).

**Table 1:** Baseline characteristics of census tracts by pharmacy desert status.

Characteristic	Overall	Non-Pharmacy Desert	Pharmacy Desert	P-value
	N = 83,398	N = 80,293	N = 3,105	
Total Population	3,743 (2,728, 4,899)	3,764 (2,750, 4,915)	3,115 (2,204, 4,354)	<0.001
Adult >65 years prop.	15.7 (11.1, 20.8)	15.7 (11.2, 20.8)	14.7 (8.7, 21.7)	<0.001
Female prop.	51.0 (49.9, 52.2)	51.0 (49.9, 52.2)	50.2 (48.9, 51.6)	<0.001
Race/Ethnicity				<0.001
White	56,216 (67%)	54,631 (68%)	1,585 (51%)	
Black	6,540 (7.8%)	6,142 (7.6%)	398 (13%)	
Hispanic	8,259 (9.9%)	7,662 (9.5%)	597 (19%)	
Asian	873 (1.0%)	870 (1.1%)	3 (<0.1%)	
AIAN	195 (0.2%)	113 (0.1%)	82 (2.6%)	
Integrated	11,315 (14%)	10,875 (14%)	440 (14%)	
Prop. Less Than High School Education	28.4 (19.8, 36.0)	28.1 (19.6, 35.7)	34.6 (28.1, 40.7)	<0.001
Prop. With No Health Insurance	7.7 (4.0, 13.7)	7.6 (3.9, 13.4)	12.3 (7.3, 19.9)	<0.001
Prop. With Ambulatory Disability	7.7 (5.0, 11.3)	7.6 (4.9, 11.1)	10.4 (6.9, 14.4)	<0.001
Median Income	62,557 (46,421, 85,836)	63,718 (47,356, 86,993)	43,203 (35,913, 50,885)	<0.001
Urbanicity				<0.001
Urban	22,546 (27%)	21,031 (26%)	1,515 (49%)	
Suburban	30,379 (36%)	30,324 (38%)	55 (1.8%)	
Rural	30,473 (37%)	28,938 (36%)	1,535 (49%)	
DDI	18.8 (14.2, 24.2)	18.6 (14.1, 23.9)	25.9 (20.4, 31.4)	<0.001
DDI – INFA	17.5 (13.5, 23.1)	17.3 (13.4, 22.8)	24.6 (17.8, 31.6)	<0.001
DDI – SE	11.5 (8.9, 14.5)	11.4 (8.8, 14.4)	14.6 (12.2, 17.3)	<0.001
Redlining index				<0.001
Neutral redlining	21,234 (25%)	20,908 (26%)	326 (10%)	
Low redlining	9,107 (11%)	9,035 (11%)	72 (2.3%)	
High redlining	20,362 (24%)	19,305 (24%)	1,057 (34%)	
Missing	32,629 (39%)	31,045 (39%)	1,650 (53%)	

Results are presented as median (IQR) for continuous and n (%) for categorical variables.

Abbreviations: Prop., proportion; DDI, digital divide index; INFA, infrastructure component; SE, socioeconomic component; AIAN, American Indian and Alaska Native.

**Fig 3:**
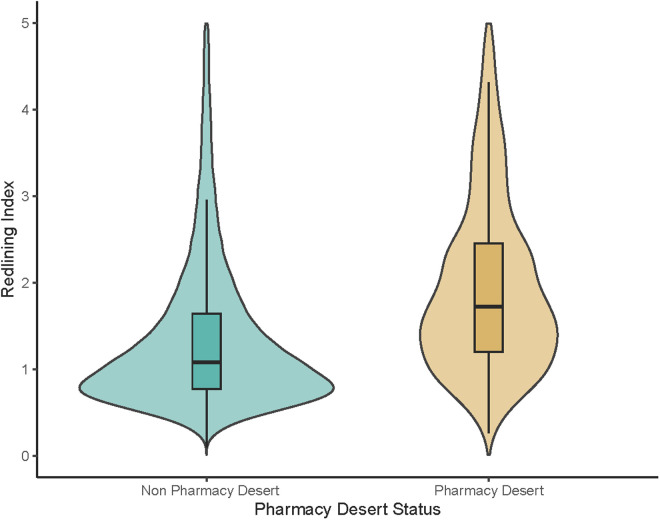
Violin plot showing the association of pharmacy desert status and redlining index.

On multivariable logistic regression analysis, census tracts with a high redlining index had a twofold increased risk of being defined as a pharmacy desert (OR 2.18, 95%CI 1.90–2.50; p < 0.001; E-value = 2.31), whereas a low redlining index was associated with reduced risk that the census tract was a pharmacy desert (OR 0.64, 95%CI 0.49–0.83; p < 0.001; E-value:1.80) (Fig 3). Furthermore, census tracts with a moderate (OR 2.84, 95%CI 2.40–3.37; p < 0.001; E-value = 2.76) or high DDI (OR 6.94, 95%CI 5.82–8.32; p < 0.001; E-value = 4.70) were much more likely to be a pharmacy desert versus census tracts with a low DDI. Moran’s I analysis demonstrated good spatial autocorrelation in the model-predicted odds of pharmacy desert classification based on DDI (Moran’s I = 0.41; p < 0.001); similarly, model-predicted odds based on the redlining index also demonstrated good spatial clustering (Moran’s I = 0.49; p < 0.001), indicating non-random spatial patterns in both redlining- and digital-related disparities. Interestingly, census tracts categorized as more segregated relative to Black (OR 2.09, 95%CI 1.74–2.51), Hispanic (OR 2.94, 95%CI 2.51–3.45), and AIAN (OR 16, 95%CI 7.88–31.2) populations were also more likely to be characterized as pharmacy deserts (all p < 0.001). Similarly, communities with a high proportion of inhabitants with less than high school education (OR 1.39, 95%CI 1.23–1.57), no health insurance (OR 1.37, 95%CI 1.21–1.55), and ambulatory disability (OR 1.30, 95%CI 1.15–1.47) were all more likely to be classified as pharmacy deserts (all p < 0.001) (S1 Table in Supporting information 1).

On secondary analysis of census tracts that met only the distance criterion and not the low-income criterion to be a pharmacy desert, similar findings were noted despite the number of census tracts classified as pharmacy deserts increasing to 8,301 (9.95%). Specifically, on multivariate analysis, census tracts with moderate (ref: low; OR 1.28, 95%CI 1.19–1.37) and high DDI (ref: low; OR 2.11, 95%CI 1.95–2.30), as well as regions with a high redlining index (ref: neutral; OR 1.38, 95%CI 1.27–1.50) were more likely to be characterized as pharmacy deserts. In contrast, census tracts with low redlining index were less likely to be characterized as pharmacy deserts (ref: neutral; OR 0.82, 95%CI 0.73–0.92) (all p < 0.001) (S2 Table in Supporting information 1). Assessment of multicollinearity revealed no concerning correlations among predictors (S3 Table in Supporting information 1).

## Discussion

Access to medications and health services is an important public health priority related to the ongoing national discussion on healthcare disparities across the US. The increasing number of pharmacy closing nationwide represent a growing obstacle for patients to access medications [5]. Moreover, pharmacies often serve as crucial points of care, offering patients a wide variety of health and prevention services such as help with complex medication regimens and drug interactions or health screenings and immunizations [3]. These roles have further expanded since the COVID-19 pandemic, during which pharmacies provided essential services such as testing and vaccinations across the country [26]. Unfortunately, major drugstore chains such as CVS and Walgreens have been closing thousands of locations nationwide in the last few years, leaving possible health gaps especially in more vulnerable communities [4]. Even though telepharmacy technologies have been proposed as a potential replacement for physical pharmacies, their implementation may be hindered in regions where access to digital technologies and internet services is still limited [27]. Additionally, pharmacy closures within urban areas may more heavily impact disadvantaged neighborhoods, such as segregated communities plagued by reduced economic development and access to resources. In this framework, the current study was important as we specifically defined the association of pharmacy deserts, digital divide, and residential redlining across the US. Specifically, we demonstrated that census tracts experiencing residential segregation had higher odds of being pharmacy deserts (OR 2.18, 95%CI 1.90–2.50; p < 0.001). Furthermore, communities experiencing a greater digital divide were more likely to be impacted by pharmacy deserts (ref: low DDI; moderate DDI: OR 2.84, 95%CI 2.40–3.37; high DDI: OR 6.94, 95%CI 5.82–8.32; both p < 0.001). These data suggest that vulnerable communities already facing economic and social challenges are being left with reduced access to pharmacy services, potentially exacerbating existing health disparities.

As timely access to medication and pharmacist intervention can improve medication adherence and health outcomes, telepharmacy services have been proposed as a possible solution to pharmacy deserts [5,28]. The effective implementation of telepharmacy services is dependent, however, on having reliable internet access and being able to use digital tools. Unfortunately, more than 42 million residents in the US lack broadband internet access, with individuals from low-income, rural, and marginalized ethnic and racial groups disproportionately impacted often as a result of internet service provider underinvestment [29]. Consequently, digital inequalities may hinder the implementation of telepharmacy, particularly among socially vulnerable populations who already face challenges such as unstable housing and significant economic hardship, potentially widening existing health inequities and limiting the benefits of telepharmacy for individuals most in need [30]. In fact, public health experts have highlighted the significant impact of regional and socioeconomic disparities in access to modern information technology (i.e., the “digital divide”) on health inequities, and therefore many experts argue that broadband internet access should be considered a key social determinant of health [31]. In a recent study that included 516,428 cancer patients treated during the COVID-19 pandemic, a greater digital divide was associated with reduced completion of video visits (OR 0.903, 95%CI 0.87–0.93; p < 0.0001) [23]. Another series reported that county-level digital divide had less influence on the likelihood of completing a surgical oncology telehealth visit during the COVID-19 pandemic, which was instead mainly influenced by age, sex, insurance status, and primary site of cancer [11]. In the current study, we measured digital divide across census tracts using the DDI, a validated measure of the availability of modern information technology. Census tracts categorized as pharmacy deserts had higher DDI, mainly because of a higher INFA component of the DDI score, highlighting the inequalities of the digital infrastructure. Furthermore, census tracts that experienced higher digital divide had a higher likelihood of being characterized as being pharmacy deserts (ref: low DDI; moderate DDI: OR 2.84, 95%CI 2.40–3.37; high DDI: OR 6.94, 95%CI 5.82–8.32; both p < 0.001). These findings suggest that the hardships faced by pharmacy desert communities may not be completely solved by implementing telehealth technologies, and are especially relevant in light of the recent termination of the Affordable Connectivity Program (ACP) in June 2024, which provided internet services and connected devices to 23 million low-income households across the US [32]. Interventions aimed at widening the implementation of broadband internet access and digital technologies in low-income communities and improving digital health literacy can help reduce health inequities that may arise from increasing pharmacy closures [33].

Neighborhoods characteristics have a significant impact on health and health inequity [34,35]. Specifically, residential segregation has been recognized as a source of different exposures and experiences based on race, which often impact health outcomes [7,18]. Historically, areas identified to be at a higher risk to default on mortgages based on their population characteristics have been targeted by redlining practices, leading to systemic disinvestment and limited access to essential services [36]. Previous studies have noted that segregated communities, as well as in low-income communities are more likely to have reduced access to healthcare facilities including pharmacies [6]. In fact, pharmacy closures not only impact rural areas in which access to pharmacies may be hindered by geographic isolation and limited infrastructure, but also affect low-income urban areas in which pharmacies are at a higher risk of closing [37]. Furthermore, lack of transportation is a major barrier to healthcare access even in urban settings, with inhabitants that do not own a car being less likely to receive timely care [38]. In the current study, we assessed the association between pharmacy deserts and contemporary redlining, measured by the redlining index which evaluated the systematic denial of mortgage financing to specific neighborhoods or applicants as a proxy for residential development and segregation. Census tracts with a higher redlining index, which reflected worse access to housing resources and environmental features such as parks and green spaces, were twice as likely to also be pharmacy deserts (OR 2.18, 95%CI 1.90–2.50; p < 0.001); in contrast, census tracts that had a lower redlining index had a lower likelihood of being pharmacy deserts (OR 0.64, 95%CI 0.49–0.83; p < 0.001). Similarly, a recent study of 13,009,569 individuals noted that residing in a redlined or in a deprived census tract was associated with 9.0% and 59.0% decreased odds of having access to a pharmacy within 1 mile, respectively. Moreover, in the most deprived neighborhoods, redlining was more strongly associated with reduced access to pharmacies with 35.0% and 51.0% lower odds of living within 1 and 2 miles of a pharmacy, respectively [36]. Defining the association of residential segregation and disparities relative to healthcare access may help identify targets for public health policies and interventions. For instance, targeted investments in underserved areas may mitigate the impact of pharmacy closures. In rural areas, expanding transportation infrastructure and incentivizing the maintenance of pharmacies may reduce patients’ geographic isolation. In urban communities, policies focused on mitigating economic disinvestment in disadvantaged areas and improving public transit access could allow patients improved pharmacy access. Suburban areas may benefit from zoning reforms and strategic placement of healthcare services. Additionally, improving transportation options in segregated neighbourhoods may help patients access more distant pharmacies.

Findings in the current study should be interpreted in light of several limitations. First, the cross-sectional design of the study limits the ability to draw causal inferences; specifically, the associations identified between redlining, digital divide, and pharmacy deserts reflect correlations at a single point in time and do not imply causation. Second, data on pharmacy locations were sourced from a publicly available source, which may contain inaccuracies. Although the strength of the association observed among AIAN communities suggests an elevated risk of pharmacy access barriers, these findings should be interpreted with caution due to the small number of relevant census tracts and the wide confidence intervals. Lastly, while pharmacy deserts were defined in accordance with previous research, no universally accepted definition of a pharmacy desert exists, possibly leading to varying results among different studies.

In conclusion, more than 10 million people across the US face increased difficulties in accessing pharmacy services. Census tracts characterized by a greater digital divide, as measured by the DDI, had higher odds to be characterized as pharmacy deserts. In these areas, the adoption of telepharmacy services alone may not sufficiently address the increasing issue of pharmacy closures. Additionally, census tracts affected by contemporary redlining had a greater risk of being pharmacy deserts, highlighting how systemic underinvestment may play a significant role in exacerbating health disparities among already disadvantaged populations. Understanding how socioeconomic and infrastructure factors influence access to healthcare resources is crucial to reduce health inequities. Efforts should be made to ensure equitable access to pharmacy services, especially for underserved populations in both rural and urban settings. Future research should be aimed at analysing temporal trends and causality, as well as evaluating the efficacy of policy interventions aimed at improving pharmacy access among disadvantaged populations.

## Supporting information

Supporting information 1 Supplementary tables. (DOCX)
