# Supplementary material for: Distribution of pharmacy deserts and its association with digital divide and residential redlining across the United States
Source: PLoS One. 2025 Aug 11;20(8):e0330027. doi: 10.1371/journal.pone.0330027 (PMC12338793; doi:10.1371/journal.pone.0330027)
Supplement: Supporting information 1 — (DOCX) [file pone.0330027.s001.docx]

**S1 Table.** Association of redlining index and digital divide index with pharmacy desert status.

| **Characteristic** | **OR** **(95% CI)** | **p-value** | **OR** **(95% CI)** | **p-value** | |
| --- | --- | --- | --- | --- | --- |
| Redlining index |  |  |  | |  |
| Neutral | ref |  | - | |  |
| Low | 0.64 (0.49 to 0.83) | **<0.001** | - | |  |
| High | 2.18 (1.90 to 2.50) | **<0.001** | - | |  |
| DDI |  |  |  | |  |
| Low | - |  | ref | |  |
| Moderate | - |  | 2.84 (2.40 to 3.37) | | **<0.001** |
| High | - |  | 6.94 (5.82 to 8.32) | | **<0.001** |
| Age ≥65 years | 0.98 (0.83 to 1.15) | 0.80 | 1.32 (1.21 to 1.45) | | **<0.001** |
| High proportion of female inhabitants (ref: low) | 0.89 (0.77 to 1.01) | 0.078 | 0.50 (0.45 to 0.55) | | **<0.001** |
| High proportion of inhabitants with less than high school education (ref: low) | 1.39 (1.23 to 1.57) | **<0.001** | 1.47 (1.35 to 1.59) | | **<0.001** |
| High proportion of inhabitants without health insurance (ref: low) | 1.37 (1.21 to 1.55) | **<0.001** | 1.43 (1.31 to 1.56) | | **<0.001** |
| High proportion of inhabitants with ambulatory disability (ref: low) | 1.30 (1.15 to 1.47) | **<0.001** | 1.14 (1.04 to 1.24) | | **0.003** |
| Race/Ethnicity |  |  |  | |  |
| White | ref |  | ref | |  |
| Black | 2.09 (1.74 to 2.51) | **<0.001** | 1.90 (1.67 to 2.17) | | **<0.001** |
| Hispanic | 2.94 (2.51 to 3.45) | **<0.001** | 2.07 (1.84 to 2.32) | | **<0.001** |
| Asian | 0.25 (0.04 to 0.76) | **0.048** | 0.12 (0.02 to 0.39) | | **0.003** |
| AIAN | 16.0 (7.88 to 31.2) | **<0.001** | 10.5 (7.68 to 14.2) | | **<0.001** |
| Integrated | 1.94 (1.65 to 2.27) | **<0.001** | 1.65 (1.47 to 1.86) | | **<0.001** |
| Results of the multivariate logistic regression analyses. P-values highlighted in bold are significant.  Abbreviations: DDI, digital divide index; AIAN, American Indian and Alaska Native. | | | | | |

**S2 Table.** Association of redlining index and digital divide index with pharmacy desert status considering census tracts that met only the distance criterion.

| **Characteristic** | **OR** **(95% CI)** | **p-value** | **OR** **(95% CI)** | **p-value** | |
| --- | --- | --- | --- | --- | --- |
| Redlining index |  |  |  | |  |
| Neutral | ref |  | - | |  |
| Low | 0.82 (0.73 to 0.92) | **<0.001** | - | |  |
| High | 1.38 (1.27 to 1.50) | **<0.001** | - | |  |
| DDI |  |  |  | |  |
| Low | - |  | ref | |  |
| Moderate | - |  | 1.28 (1.19 to 1.37) | | **<0.001** |
| High | - |  | 2.11 (1.95 to 2.30) | | **<0.001** |
| Age ≥65 years | 0.91 (0.82 to 1.00) | 0.051 | 1.37 (1.29 to 1.45) | | **<0.001** |
| High proportion of female inhabitants (ref: low) | 0.60 (0.55 to 0.66) | **<0.001** | 0.34 (0.31 to 0.36) | | **<0.001** |
| High proportion of inhabitants with less than high school education (ref: low) | 1.39 (1.29 to 1.51) | **<0.001** | 1.56 (1.48 to 1.65) | | **<0.001** |
| High proportion of inhabitants without health insurance (ref: low) | 0.91 (0.83 to 0.99) | **0.032** | 0.97 (0.92 to 1.03) | | 0.40 |
| High proportion of inhabitants with ambulatory disability (ref: low) | 0.88 (0.81 to 0.96) | **0.005** | 0.85 (0.80 to 0.90) | | **<0.001** |
| Race/Ethnicity |  |  |  | |  |
| White | Ref |  | ref | |  |
| Black | 1.16 (1.01 to 1.33) | **0.038** | 1.00 (0.90 to 1.12) | | 0.97 |
| Hispanic | 1.64 (1.47 to 1.83) | **<0.001** | 1.18 (1.08 to 1.28) | | **<0.001** |
| Asian | 1.49 (1.10 to 1.97) | **0.007** | 0.96 (0.74 to 1.22) | | 0.74 |
| AIAN | 9.04 (4.61 to 17.4) | **<0.001** | 5.15 (3.81 to 6.95) | | **<0.001** |
| Integrated | 1.35 (1.23 to 1.49) | **<0.001** | 1.12 (1.04 to 1.21) | | **0.002** |
| Results of the multivariate logistic regression analysis. P-values highlighted in bold are significant.  Abbreviations: DDI, digital divide index; AIAN, American Indian and Alaska Native. | | | | | |

**S3 Table.** Variance Inflation Factor of all covariates in the multivariable models assessing the relationship of redlining index and digital divide index with pharmacy desert status.

| **Variable** | **Model 1 VIF** | **Model 2 VIF** |
| --- | --- | --- |
| Redlining index | 1.06 | - |
| DDI | - | 1.09 |
| Age ≥65 years | 1.06 | 1.12 |
| High proportion of female inhabitants | 1.15 | 1.10 |
| High proportion of inhabitants with less than high school education | 1.08 | 1.08 |
| High proportion of inhabitants without health insurance | 1.13 | 1.14 |
| High proportion of inhabitants with ambulatory disability | 1.09 | 1.13 |
| Race/Ethnicity | 1.07 | 1.06 |

Abbreviations: DDI, digital divide index; VIF, Variance Inflation Factor.
